# Variations in Mating and Reproduction in Oriental Fruit Moth Caused by Adult Physiological State in Laboratory Tests

**DOI:** 10.3390/insects15060457

**Published:** 2024-06-18

**Authors:** Weina Kong, Yi Wang, Na Li, Weiye Cao, Xuefeng Hu, Changnian Liu, Guofei Niu, Jie Li, Ruiyan Ma

**Affiliations:** 1 College of Plant Protection, Shanxi Agricultural University, Taiyuan 030031, China; kwnlll@126.com (W.K.); wangyiggg@126.com (Y.W.); li2549205899@163.com (N.L.); ksmteidy@126.com (W.C.); xuefenghu2021@126.com (X.H.); liucn0807@163.com (C.L.); rlnu770313@163.com (G.N.); 2 Shanxi Agricultural University, Taiyuan 030031, China

**Keywords:** *Grapholita molesta*, adult physiological status, mating preference, reproductive system, offspring production

## Abstract

**Simple Summary:**

Due to a combination of monandry and promiscuity in *Grapholita molesta* (Busck), this study investigated the interaction of different adult ages (3, 5, or 7 days) and mating history (unmated or mated) in each sex on the mating selection, development of the reproductive system, and offspring production in the laboratory. The results show that the physiological status of females may be responsible for mating and reproductive decisions, and the physiological status of males may regulate the ranges in behavioral changes. As a species with high reproductive potential, the presence of males accelerated the growth of the *G*. *molesta* populations. Our results would provide theoretical data to further understand the reproductive biology of *G. molesta* in the field.

**Abstract:**

*Grapholita molesta* (Busck) is a pest of rosaceous fruit plants worldwide. Due to a combination of monandry and promiscuity in *G. molesta*, the age and mating history of both sexes significantly affected the mating and reproductive success. In this study, the interactions of different ages (3, 5, or 7 days) and mating history (unmated or mated) in each sex on the mating selection, reproductive system, and offspring production were investigated in the laboratory. The results showed that these differences mainly occurred in young females or males, associated with unmated or mated state. Especially, the 3-day-old unmated females were preferred by the 7-day-old males but discriminated against by the 3- or 5-day-old unmated males, whereas the 3-day-old mated males were preferred by the 3-day-old mated or 7-day-old females but discriminated against by the 3- or 5-day-old unmated females. The lengths of the ovarian ducts were affected by age in the unmated females, with the greatest length being found at 7 days old. The size of testes varied with age in the unmated males, being the largest at 3 days old. At 3 days old, the testes size of the unmated males was larger than that of the mated males. The pairing of 5-day-old unmated females × 3-day-old mated males maximized the successful matings. The least productive pairing was 7-day-old unmated females × 5-day-old mated males. The pairing of 5-day-old mated males × 3-day-old mated females had the lowest number of matings and the highest number of offspring. The pairing of 3-day-old mated females × 3-day-old mated males had a high rate of mating success and the most offspring. These results revealed the different roles between females and males because of physiological states in terms of the reproductive biology in *G. molesta*.

## 1. Introduction

*Grapholita molesta* (Busck) (Lepidoptera: Tortricidae) is a worldwide orchard pest and is a serious problem in the fruit-growing regions of China [1]. It attacks almost all rosaceous crop species, including the species of *Prunus*, *Malus*, and *Pyrus*, from the “green tip” stage up to harvest, over a 4-to-9-month period [2]. Over the growing season, the infestation site of *G. molesta* on the plant changes, from attacks by first-generation larvae on sprouting shoots in spring and early summer to feeding by larvae of the summer generations inside the fruit [3]. Until recently, this pest has been primarily controlled by the use of one or more broad-spectrum insecticides [4]. The issues associated with the heavy and repeated use of insecticides, including insecticide resistance, toxicity to natural enemies, and food residues, have provided an impetus for the research and development regarding new control technologies [5].

Sex pheromones are used for monitoring, mass trapping, and mating disruption and are effective supplements to pesticides against *G. molesta* [6]. The value of using sex pheromones may depend on the biological details of the target pest, including the adult reproductive status, mating system, population density, mobility, etc. [7]. The features of *G. molesta*, such as a male-biased sex ratio, multiple mating by males, and single mating by females, made it more likely that the use of sex pheromones will increase control costs and have limited effectiveness [8]. However, most of the previous studies have reported that multiple mating in insects can significantly reduce the efficacy of sex pheromones for managing field populations [9]. Thus, it is very important to understand the reproductive biology of *G. molesta* for the improvement and optimization of sex pheromone technology.

Mating systems have a great impact on the reproductive output of insects [10]. Because of the asymmetry in the number of partners in certain mating systems, an individual’s fitness is a function of both its own mating strategy and its partner’s mating strategy [11]. In the case of polyandry, the ability of a portion of the males to monopolize the access to the females is thought to be the fundamental determinant of such mating systems [12], whereas the female’s investment in reproduction is dictated by her physiological state, the expectation of future reproductive opportunities, and the trade-off between the two [13]. We reported earlier that, under laboratory conditions, the male *G. molesta* preferred young females, whereas the females preferred mated males [14]. These observations suggest that the mating system of *G. molesta* is modulated by the age and mating history of both sexes, which may be related to the development of the female ovaries and male ductus among the mating and egg-maturation and oviposition stages [15,16]. However, when one sex encounters the opposite sex based on different physiological states in this type of mating system, the variations in their mating and reproductive behavior because of individual fitness are unclear.

Here, we investigated the effects of age and mating history between the two sexes on the mate preference, reproductive system, totally successful mating, and fertile egg production in laboratory tests. The determination of the plastic degree in the mating system of *G. molesta* will help to clarify the reproductive biology in order to improve the efficacy of sex pheromone control technology.

## 2. Materials and Methods

### 2.1. Insect Rearing

*G. molesta* used in our experiments were from a colony established with larvae collected from infested orchards (Taigu, Shanxi, China) in 2010 and maintained for >80 generations. Approximately 30% of this laboratory population was renewed each year with new larvae collected from the same orchards. To accomplish this, larvae from infested shoots or fruits were collected and taken to the laboratory, removed from the plant material, and placed in glass tubes (3.6 cm diameter × 8 cm high). These tubes were filled with an artificial diet [17], plugged with absorbent cotton balls, and held until larvae pupated. Larvae were reared at 26 ± 0.8 °C, 75 ± 5% RH, and a photoperiod of 15:9 (L:D) h, following the protocol of Kong et al. [18]. We observed no difference in adult emergence, mating, and reproductive performance between laboratory and wild strains [8]. Moths used in experiments were collected on the morning of their emergence and were designated on that day as 1-day-old moths.

### 2.2. Experimental Designs Applicable to All Experiments

Unmated or mated females and males, of 3 different ages (3, 5, or 7 days old) were used in all experiments. A single unmated female or male at 1–2 days old was individually paired with the opposite sex of the same age in a mating cage, and then was isolated in separate cage until the required age states of the experiments were obtained. Especially, the number of eggs laid by mated females was recorded daily before paring in Exp.#3. Courtship bouts were observed from the initial approach of a male until either copulation began, the female flew away, or 30 min had elapsed. Copula was judged to have occurred if wings of a male were under wing of a female, and male’s antennae were placed on the female’s back [19]. All observations were conducted from 5 p.m. to 9 p.m., which was the time of maximal calling by females, and a diode red light emitter was used for insect observation during the scotophase [14]. All experiments were conducted from 2020 to 2023.

#### 2.2.1. Exp. #1. Mating Selection

The behavioral responses of one sex to the opposite sex based on six physiological states in *G. molesta* were evaluated in a six-arm olfactometer in agreement with the method reported in Cao et al. [20,21]. Briefly, the six-arm olfactometer consisted of a central chamber (9.5 cm internal diameter, 7.5 cm high) with six arms (6 cm length, 1.5 cm internal diameter), each connected to a glass tube (20 cm length and 1.5 cm diameter) that projected outwards at an equidistance, with 60° angles between pairs of flasks. Each arm was connected through Teflon tubing to a triangular glass bottle (250 mL), which was used to contain test stimuli. The airflow was set at 200 mL·min^–1^ to drive the odor source to test moths. Previous reports show that male hairpencil components during courtship attract sex-pheromone-releasing females from several centimeters [22]. One sex with each of six physiological states in *G. molesta* was introduced in groups (1 individual per group) with a brush, and six physiological states of the opposite sex were used as six test stimuli. Moths that entered an arm of the olfactometer within 20 min were counted as having made a choice for a particular odor source. Moths that did not enter an arm within this time were considered ‘non-responders’. After each test, the olfactometer was cleaned, dried, and the arms were rotated (60°). Bioassays were replicated 12 to 14 times and were carried out between 17:00 p.m. and 21:00 p.m. A red light was also placed in the center, 60 cm above the chamber, to eliminate any light bias. Before the beginning of the olfactometer assays, the system was cleaned with ethanol (95%) and rinsed with distilled water. The olfactometer system was placed in a controlled temperature room held at 25 ± 2 °C. The selectivity rate was calculated as Equation (1).

(1) $$Selective\;rate\left(\%\right)=\frac{Adult\;number\;with\;behavioral\;responses}{Total\;number\;of\;test\;adults}\times 100\%$$

#### 2.2.2. Exp. #2. Anatomical and Morphological Observations of Reproductive System

After cocooning, fifty male pupae and fifty female pupae were selected randomly and held for adult emergence. Moths of known age could be dissected to observe the morphology of their reproductive systems according to the report by Zhang et al. [16]. For dissections, unmated or mated moths were chosen for each of 3 ages (3, 5, and 7 days). Moths were dissected in a glass Petri dish (90 mm diameter) (Renyuan Company, Cangzhou, China) containing phosphate buffered saline (PBS) buffer (Shenggong Biological Engineering Co., Ltd., Shanghai, China) using a Leica stereomicroscope M205C (Shanghai Baihe Instrument Technology Co., Ltd., Shanghai, China). A tip tweezer (Shenggong Biological Engineering Co., Ltd., Shanghai, China) was used to hold the thoracic-abdominal junction of the moth, and another tip tweezer was used to remove the cephalothorax. The tweezers were then used to open the abdominal epidermis along the thoracic-abdominal junction to reveal the abdominal cavity. The ovary or testes were then collected, removing other surrounding tissues [23]. Ovaries and testes were transferred to a glass slide with a drop of PBS buffer. The glass slide was placed under a Leica DFC450 digital camera (Dayueweijia Science and Technology Ltd., Beijing, China) attached to a Leica stereomicroscope M205C, and the ovaries or testes were photographed. Either five females or five to six males were examined for each mating state. We recorded the lengths of ovarian ducts and the long and short diameters of testes in order to evaluate their development [24]. The size of testes was calculated using Equation (2) [25].

(2) $$Size\;of\;tests=\frac{\pi \times long\;diameter\times short\;diameter}{4}$$

#### 2.2.3. Exp. #3. Mating and Reproductive Traits

The integrated effects of varying age and mating history of males and females on mating and reproductive success were assessed in one experiment with three setting conditions (SCs) as follows.

In SC 1, a single 3-day-old virgin or mated female was individually paired with a single virgin or mated male of 3 ages (3, 5, or 7 days old).

In SC 2, a single 5-day-old virgin or mated female was individually paired with a single virgin or mated male of 3 ages (3, 5, or 7 days old).

In SC 3, a single 7-day-old virgin or mated female was individually paired with a single virgin or mated male of 3 ages (3, 5, or 7 days old).

All pairings were performed individually in transparent mating cages (1.5 L bottles cut along the center; 15 cm diameter, 20 cm high) with absorbent cotton saturated daily with a 5% sugar-water solution. We observed and recorded the start and end times of each copulation, number of the day on which copulation occurred, and total number of copulations. The number of eggs laid on the surface of mating cage was recorded daily, and each egg was marked with a small circle outside mating cage using a marker pen until the female died. It normally takes three days for most fertile eggs to hatch. After this time, eggs were considered fertilized if they were darker in color [18]. The number of fertile eggs (those developing to the blackhead stage) vs. infertile eggs (no darkening) were counted during the three-day observation period. The preoviposition, oviposition, and post-oviposition periods of females were recorded until they died. For males and females that died before any egg was laid, only the lifespan was recorded [8]. All containers were maintained in a growth chamber under the same conditions as the colony. If a test female or male died during the course of the experiment, the replicate was discarded. Females mated once in their lifetime and laid eggs the same day they mated [8]. Of the 233 couples observed of this study, 100% (*n* = 233) of the pairs mated once; 24.03% (*n* = 56) of the pairs mated twice; 8.58% (*n* = 20) of the pairs mated three times; 4.29% (*n* = 10) of the pairs mated four times; 2.15% (*n* = 5) of the pairs mated five times; 0.43 (*n* = 1) of the pairs mated six times. So, oviposition information of females with mated state after the mating test was not available. However, we just wanted to know the effects of mating on oviposition by mated females.

All treatments under each of SCs were repeated at least six times because of high mortality rate of old moths. Overall, three-hundred-forty-nine cages were set up, and three-hundred-forty-nine males and three-hundred-forty-nine females were used for these observations.

### 2.3. Statistical Analysis

All data sets were square root transformed to stabilize their variances before analysis. For data from selection tests, the selective rate of one sex within each of six physiological states to the opposite sex in six physiological states was analyzed using chi-squared tests. For virgin or mated states, both the lengths of ovarian ducts of females and size of testes of males at 3 ages were analyzed with one-way ANOVA. Post hoc comparisons of means were conducted with Tukey’s multiple range tests. For the two morphological indicators, significant differences between the virgin and mated states for each of both sexes within each of 3 ages were analyzed using independent sample tests. Different *t*-tests for equality of the means were used when equal variances could be assumed (Levene’s test for equality of variances, *p* > 0.05) or not (Levene’s test for equality of variances, *p* < 0.05). When one sex within each of six physiological states was individually paired with the opposite sex within each of six physiological states, the number of matings per cage, duration of matings per cage, number of eggs laid by a female per cage after pairing, percentage of eggs hatched per cage after pairing, total number of eggs laid by a female per cage, and total percentage of eggs hatched per cage among eighteen pairing combinations within each of two mating histories for each of both sexes were analyzed with one-way ANOVA. Post hoc comparisons of means were conducted with Tukey’s multiple range tests.

## 3. Results

### 3.1. Effect of Adult Physiological States on Selection of One Sex for the Opposite Sex

**Figure 1:**
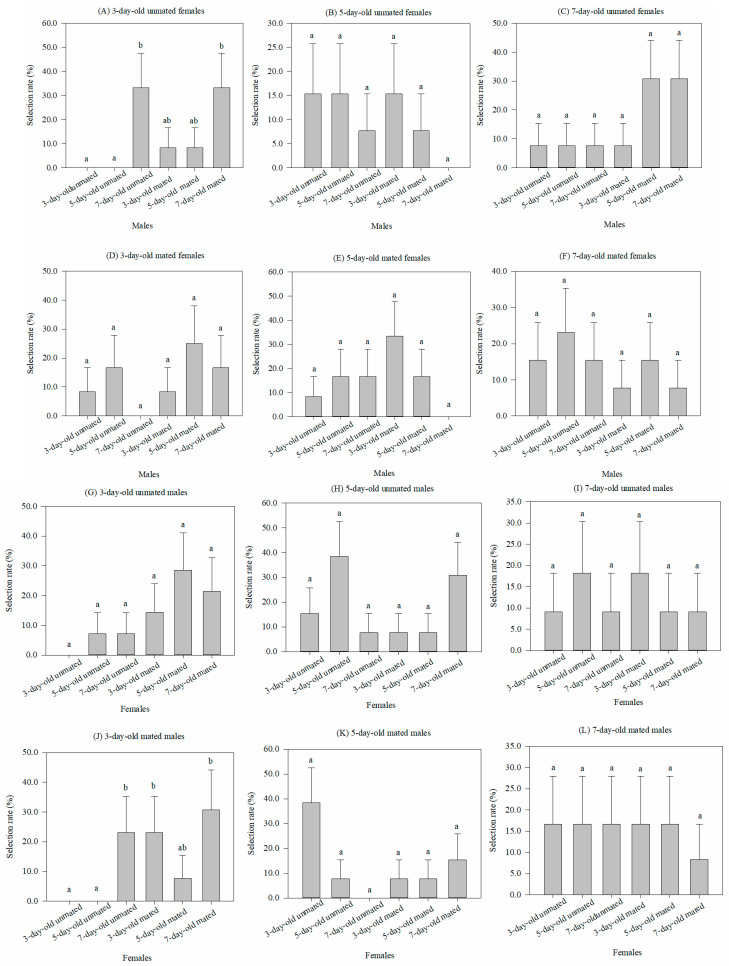
Comparison of the selective rates (%) (mean ± SEM) of one sex of *Grapholita molesta* in 6 physiological states with the opposite sex, which also has 6 physiological states. Different lowercase letters indicate significant differences among physiological states of one sex within each physiological state of the opposite sex at *p* < 0.05, based on Turkey’s multiple-range test.

When males of six physiological states (3 ages × mated vs. unmated) were selected by females within each of six states (3 ages × mated vs. unmated), the male conditions had no significant effect on the rates for five of the six female conditions. No significance was found for either the 5-day-old (*χ*^2^ = 3.997, df = 5, *p* = 0.55) or 7-day-old (*χ*^2^ = 6.674, df = 5, *p* = 0.246) virgin females, or the 3-day-old (*χ*^2^ = 5.364, df = 5, *p* = 0.373), 5-day-old (*χ*^2^ = 6.959, df = 5, *p* = 0.224), or 7-day-old (*χ*^2^ = 1.83, df = 5, *p* = 0.872) mated females (Figure 1B–F). Only for the 3-day-old virgin females was there any significance among the male states, with selection having the highest rate for the 7-day-old males (*χ*^2^ = 13.703, df = 5, *p* < 0.05) (Figure 1A).

Conversely, there was no significant difference among the selective rates of six physiological states of females for either the 3-day-old (*χ*^2^ = 8.024, df = 5, *p* = 0.155), 5-day-old (*χ*^2^ = 7.729, df = 5, *p* = 0.172), or 7-day-old (*χ*^2^ = 1.082, df = 5, *p* = 0.956) virgin females (Figure 1G–I), or 5-day-old (*χ*^2^ = 10.103, df = 5, *p* = 0.072) or 7-day-old (*χ*^2^ = 0.608, df = 5, *p* = 0.988) mated females (Figure 1K–L). However, the selective rates by the 3-day-old mated males were higher for either the 7-day-old virgin females or 3-day-old and 7-day-old mated females when compared to the 3-day-old or 5-day-old virgin females, but their rates did not differ from that of the 5-day-old mated females (*χ*^2^ = 12.274, df = 5, *p* < 0.05) (Figure 1J).

### 3.2. Effect of Adult Physiological States on Reproductive System

**Figure 2:**
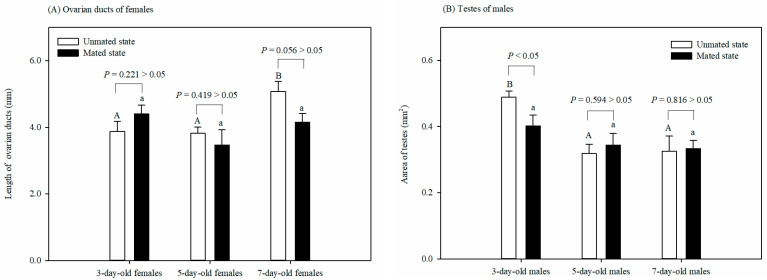
Effect of 6 physiological states on the lengths of ovarian ducts of females (**A**) and the testes size of males (**B**) in *Grapholita molesta* (mean ± SEM). Different uppercase or lowercase letters indicate significant differences among ages within each sex on unmated or mated states at *p* < 0.05, based on Turkey’s multiple-range test. There was a significant or non-significant difference in either ovarian ducts or testes between the unmated and mated females within each age group at a *p* < 0.05 or *p* > 0.05 level, based on Levene’s test for equality of variances.

The length of the ovarian ducts of the mated females was not affected by age (*F* = 1.962, df = 2, 14, *p* = 0.183). However, the length of the ovarian ducts of the 7-day-old unmated females was significantly longer than that of the 3-day-old or 5-day-old unmated females (*F* = 6.921, df = 2, 15, *p* < 0.05). There was no significant difference in the length of the ovarian ducts between the virgin and mated females at any age: 3 (*t* = −1.326, df = 8, *p* = 0.221), 5 (*t* = 0.846, df = 9, *p* = 0.419), or 7 (*t* = 2.236, df = 8, *p* = 0.056) days old (Figure 2A).

The testes size of the mated males was not affected by age (*F* = 1.39, df = 2, 14, *p* = 0.286). However, the testes size of the 3-day-old unmated males was larger than that of the 5-day-old or 7-day-old unmated males (*F* = 7.827, df = 2, 14, *p* < 0.05). There was no significant difference in testes size between the unmated and mated males at 5 (*t* = −0.554, df = 8, *p* = 0.594 > 0.05) or 7 (*t* = −0.24, df = 8, *p* = 0.816 > 0.05) days old. The tests size of the unmated males was, however, larger than that of the mated males at 3 days old (*t* = 0.237, df = 8, *p* < 0.05) (Figure 2B).

### 3.3. Effect of Adult Physiological States on Mating and Reproductive Success

When 3, 5, or 7-day-old unmated females were paired with six physiological states of males, there were significant differences among the cages with the six physiological states of males in (1) the number of matings per cage (*F* = 2.134, df = 17, 170, *p* < 0.05), (2) number of eggs laid by a female per cage (*F* = 5.942, df = 17, 170, *p* < 0.05), and (3) percentage of eggs hatched per cage (*F* = 2.238, df = 17, 170, *p* < 0.05), but not in the duration of matings per cage (*F* = 1.516, df = 17, 170, *p* = 0.095) (Table 1).

When 3-, 5-, and 7-day-old mated females were paired with six physiological states of males, there were significant differences among the cages with six physiological states of males in (1) the number of eggs laid by a female per cage after pairing (*F* = 9.633 df = 17, 177, *p* < 0.05), (2) percentage of eggs hatched per cage after pairing (*F* = 2.366, df = 17, 177, *p* < 0.05), and (3) total percentage of eggs hatched per cage (*F* = 2.401, df = 17, 177, *p* < 0.05), but not in the number of matings per cage (*F* = 1.423, df = 17, 177, *p* = 0.132), duration of matings per cage (*F* = 1.362, df = 17, 177, *p* = 0.162), or total number of eggs laid by a female per cage (*F* = 1.659, df = 17, 177, *p* = 0.056) (Table 2).

When 3-, 5-, and 7-day-old virgin males were paired with six physiological states of females, there were significant differences among the cages with six physiological states of females in (1) the number of matings per cage (*F* = 10.698, df = 17, 176, *p* < 0.05), (2) duration of matings per cage (*F* = 7.361, df = 17, 176, *p* < 0.05), (3) number of eggs laid by a female per cage after pairing (*F* = 10.74, df = 17, 176, *p* < 0.05), and (4) total number of eggs laid by a female per cage (*F* = 9.535, df = 17, 176, *p* < 0.05), but not in the percentage of eggs hatched per cage after pairing (*F* = 1.665, df = 17, 176, *p* = 0.055) or total percentage of eggs hatched per cage (*F* = 1.685, df = 17, 176, *p* = 0.05) (although nearly so) (Table 3).

When 3-, 5-, and 7-day-old mated males were paired with six physiological states of females, there were significant differences among the cages with six physiological states of females in (1) the number of matings per cage (*F* = 6.631, df = 17, 171, *p* < 0.05), (2) duration of matings per cage (*F* = 5.388, df = 17, 171, *p* < 0.05), (3) number of eggs laid by a female per cage after pairing (*F* = 2.944, df = 17, 171, *p* < 0.05), (4) percentage of eggs hatched per cage after pairing (*F* = 2.86, df = 17, 171, *p* < 0.05), (5) total number of eggs laid by a female per cage (*F* = 2.631, df = 17, 171, *p* < 0.05), and (6) total percentage of eggs hatched per cage (*F* = 2.72, df = 17, 171, *p* < 0.05) (Table 4).

## 4. Discussion

In *G*. *molesta*, mating occurs throughout the entire adult life stage, and the first mating can occur as early as the first night after adult emergence, at which time females can also produce fertile eggs [8].

Our results showed that the selectivity of either unmated females except for 3 days old or mated females to males was not affected by the physiological states of males, whereas the selectivity of either mated males except for 3 days old or unmated males to females was not related with the physiological states of females, which may be related with a difference in the mating number and age-delayed mating between females and males [14]. The 3-day-old unmated females were favored by the older males but were discriminated against by the young and middle-aged males. In contrast, the 3-day-old mated males were favored by either the young mated females or old females but were discriminated against by either the young or middle-aged unmated females. For most insects, old adults prefer young states of the opposite sex for mating [26,27]. In the observations, the males preferred the unmated females and the females preferred the mated males, which was consistent with our previous report on the multiple and repeated mating of both sexes in *G*. *molesta* [14]. For insects in which one sex produces a sex pheromone that attracts the opposite sex, age-dependent olfactory plasticity is also linked to sexual maturation [28]. Mated males do not respond behaviorally to the female sex pheromone as compared with that of unmated males because of a fast-acting, transient neuronal plasticity that ‘switches off’ the olfactory system, which could prevent males from mating [29]. These results suggest that the pheromone-guided behavior may be more modulated by age than mating history.

For the development of the reproductive systems of male and female *G*. *molesta* in different physiological states, our results showed that age did not affect the length of the ovarian ducts of the mated females, and mating did not affect the length of the ovarian ducts of females of the same age. The aspects are supported by the report of Shukla et al. [30] and may be related to our daily feeding of honey to supplement nutrition [31]. However, some studies report that the development of the ovarian system was much slower in unmated females than in mated females [31]. Other studies report that female mating accelerates the rate of ovariole development [32,33]. The disruption of the deposition of yolk protein in the ovary and the shortened lengths of the ovarian ducts resulted in reduced female fecundity [24]. Furthermore, age in our study also affected the length of the ovarian ducts of the unmated females, and the length of the ovarian ducts in the unmated females was greatest at 7 days of age. Ovarioles grow continuously during ovarian development [34,35]. For males, we observed that age affected the testes size of the unmated moths, and the size was greatest at 3 days of age. Also, mating did not affect the size of male testes at 5 or 7 days of age, but, at 3 days of age, the testes of the unmated males were larger than the mated males. For the mated males, age did not affect the testes size. The testes decrease in volume as sperm bundles are transferred from the testes to the duplex where the spermatophore is formed, which is then transferred to the female during copulation [36,37]. For *G*. *molesta*, the restoration of sperm content begins within 6 h and is fully restored within 18 h, after which males can mate again [16]. Our findings suggest that age may induce ovary development, while testes restoration is responsive to mating.

Our comparative results showed that either the post-mating and lifetime offspring production of the females or the number of matings of the unmated females was affected by the physiological status of the male sires. Inversely, the physiological status of the females affected the mating success of the males, the post-mating and lifetime offspring production sired by males, and the post-mating percentage of hatched eggs linked to mated males. Many studies have reported that mating can stimulate female fecundity and fertility [38,39,40]. Because female *G*. *molesta* seldom mate more than once, the delayed mating of females does not affect the daily oviposition rate [8,41]. Because male *G*. *molesta* can copulate many times, the male mating history is inversely proportional to the spermatophore production but has no effect on the egg production by female partners [8,42]. The above results suggest that the effect of the physiological state of males on unmated females may be greater than that on mated females, but the physiological status of females had a significantly higher effect on mated males than on unmated males.

Specifically, when paired with unmated females, the mating success of males was enhanced, but the number of offspring sired by the males decreased. In contrast, when males were paired with mated females, males’ mating success was lower, but the number of offspring sired by the males increased. The states for either unmated females paired with males or mated males paired with females that seemed best in mating success were 5-day-old unmated females and 3-day-old mated males. The males discriminated against the ≥5-day-old females in offspring production, but the females preferred once-mated males in terms of mating opportunity [14]. The 3-day-old mated females and 3-day-old mated males produced the highest number of offspring among the combination of the unmated females paired with males and had success in mating among the combination of the unmated males paired with females. The 5-day-old mated females and 3-day-old mated males had the highest number of offspring despite unsuccessful mating among the combination of the mated males paired with females. The preference of the 3-day-old mated males for young or middle-aged mated females was most efficient for mating opportunity and offspring production at that time, and this is why the differences occur [42]. The state of the 7-day-old unmated females and 5-day-old mated males had the lowest number of offspring for either unmated females paired with males or mated males paired with females. This resulted from the age-delayed mating of females, which is associated with significantly lower fecundity and fertility in older females [43]. These findings suggest that the presence of males is conducive to the mating of the unmated females and to the reproduction of the mated females.

The results obtained in this study indicate that encounters of young mated males with either young unmated females or young mated females may cause high mating or oviposition rates, respectively. These results characterize *G*. *molesta* as a species with high reproductive potential, and the presence of the mated males accelerated the growth of the *G*. *molesta* populations, which is an important trait to consider regarding the possibility of improving the integrated pest management of *G*. *molesta*, particularly in the practical application of pheromones. Control strategies might be suggested such that young mated males should be disrupted and trapped by sex pheromones of young unmated females in order to avoid the conversion of young females with an unmated state to a mated state for decreasing the mating and reproductive opportunities. However, because many natural and non-natural factors can be present in the field, it should be noted by other authors that the results obtained from laboratory tests are unlikely to be reflected in the field. In fact, for optimizing the application of pheromones in the field, it is better to clarify how female and male moths respond to sex pheromones directly as a function of age and mating status in *G*. *molesta* and analyze the spatio-temporal distributions of different ages and sexes of insect targets during the relevant seasonal windows in the future.

## Figures and Tables

**Table 1:** The number of matings per cage, duration of matings per cage, number of eggs laid by a female per cage, and percentage of eggs hatched per cage from males in one of six physiological states paired with an unmated female within each age group in the laboratory.

Unmated Female	Male		Number of Matings per Cage	Duration of Matings per Cage (min)	Number of Eggs Laid by a Female per Cage	Percentage of Eggs Hatched per Cage (%)
3-day-old	3-day-old	Mated	2.40 ± 0.45 ab	63.20 ± 11.90 a	135.80 ± 18.28 d	61.73 ± 10.81 ab
		Unmated	1.10 ± 0.10 ab	26.60 ± 3.93 a	37.70 ± 14.95 abc	81.02 ± 10.30 ab
	5-day-old	Mated	1.00 ± 0.19 a	31.50 ± 5.45 a	91.25 ± 10.16 bcd	82.74 ± 3.67 ab
		Unmated	1.60 ± 0.40 ab	55.30 ± 12.92 a	93.00 ± 25.47 abcd	95.95 ± 2.46 ab
	7-day-old	Mated	1.33 ± 0.37 ab	33.11 ± 8.20 a	125.44 ± 18.88 d	79.03 ± 7.79 ab
		Unmated	1.10 ± 0.10 ab	34.70 ± 2.26 a	128.30 ± 21.51 d	96.48 ± 1.59 b
5-day-old	3-day-old	Mated	2.50 ± 0.56 b	68.40 ± 15.49 a	134.90 ± 14.37 d	78.32 ± 8.94 ab
		Unmated	1.30 ± 0.15 ab	35.50 ± 4.27 a	124.90 ± 10.54 d	98.00 ± 0.79 b
	5-day-old	Mated	1.80 ± 0.36 ab	50.60 ± 9.85 a	144.40 ± 20.60 d	84.76 ± 5.10 b
		Unmated	1.00 ± 0.00 ab	35.00 ± 4.69 a	25.67 ± 11.14 ab	76.63 ± 11.73 ab
	7-day-old	Mated	1.20 ± 0.13 ab	39.70 ± 4.00 a	114.10 ± 18.45 cd	83.81 ± 6.85 ab
		Unmated	1.50 ± 0.27 ab	46.60 ± 6.56 a	107.80 ± 19.42 cd	97.16 ± 1.20 b
7-day-old	3-day-old	Mated	1.30 ± 0.15 ab	36.80 ± 5.61 a	100.80 ± 13.67 cd	62.29 ± 11.88 ab
		Unmated	1.10 ± 0.10 ab	30.90 ± 2.96 a	94.00 ± 17.55 bcd	86.90 ± 7.40 b
	5-day-old	Mated	1.60 ± 0.31 ab	47.30 ± 11.81 a	69.10 ± 19.91 abcd	49.96 ± 14.00 a
		Unmated	1.00 ± 0.00 ab	35.90 ± 4.07 a	68.10 ± 18.62 abcd	81.41 ± 8.79 ab
	7-day-old	Mated	1.17 ± 0.40 ab	34.67 ± 13.33 a	121.00 ± 22.86 d	76.21 ± 8.38 ab
		Unmated	1.00 ± 0.00 ab	42.33 ± 6.68 a	13.00 ± 4.18 a	77.56 ± 7.90 ab

Mean ± standard error followed by the same letter in the column do not differ by Turkey’s multiple-range test (*p* ≥ 0.05).

**Table 2:** The number of matings per cage, duration of matings per cage, number of eggs laid by a female per cage after pairing, percentage of eggs hatched per cage after pairing, total number of eggs laid by a female per cage, and total percentage of eggs hatched per cage from males in one of six physiological states paired with a mated female within each age group in the laboratory.

Mated Female	Male		Number of Matings per Cage	Duration of Matings per Cage (min)	Number of Eggs Laid by a Female per Cage after Pairing	Percentage of Eggs Hatched per Cage after Pairing (%)	Total Number of Eggs Laid by a Female per Cage	Total Percentage of Eggs Hatched per Cage (%)
3-day-old	3-day-old	Mated	0.50 ± 0.31 a	12.90 ± 7.16 a	141.10 ± 12.31 def	94.38 ± 1.04 c	148.20 ± 14.22 a	94.08 ± 1.10 b
		Unmated	0.50 ± 0.17 a	15.80 ± 6.27 a	170.00 ± 14.54 ef	91.25 ± 1.39 bc	170.60 ± 14.71 a	90.99 ± 1.45 b
	5-day-old	Mated	0.30 ± 0.15 a	11.60 ± 6.57 a	171.00 ± 12.75 f	92.15 ± 1.89 bc	175.10 ± 12.61 a	89.87 ± 1.74 b
		Unmated	0.50 ± 0.17 a	22.90 ± 8.31 a	170.70 ± 13.35 f	71.09 ± 10.43 abc	170.80 ± 13.34 a	71.10 ± 10.43 ab
	7-day-old	Mated	0.22 ± 0.15 a	6.33 ± 4.26 a	129.56 ± 19.79 def	88.31 ± 5.85 bc	134.44 ± 20.10 a	87.96 ± 5.40 b
		Unmated	0.50 ± 0.17 a	18.50 ± 6.27 a	131.90 ± 9.77 def	91.73 ± 2.53 bc	134.00 ± 9.61 a	90.15 ± 2.47 b
5-day-old	3-day-old	Mated	0.10 ± 0.10 a	3.70 ± 3.70 a	89.90 ± 14.29 bcd	90.53 ± 2.34 bc	119.20 ± 14.98 a	75.67 ± 6.86 ab
		Unmated	0.20 ± 0.13 a	5.90 ± 3.93 a	94.70 ± 9.75 bcde	88.24 ± 3.19 bc	119.90 ± 12.44 a	87.98 ± 2.46 b
	5-day-old	Mated	1.20 ± 0.47 a	52.70 ± 30.46 a	120.90 ± 7.64 def	81.58 ± 6.91 abc	161.60 ± 11.62 a	82.06 ± 5.41 b
		Unmated	0.10 ± 0.10 a	6.00 ± 6.00 a	107.30 ± 9.81 cdef	89.44 ± 3.36 bc	135.80 ± 10.24 a	87.67 ± 3.38 b
	7-day-old	Mated	0.40 ± 0.22 a	15.10 ± 8.29 a	89.90 ± 14.38 abcd	62.50 ± 11.15 a	118.40 ± 18.80 a	59.81 ± 10.13 a
		Unmated	0.20 ± 0.13 a	8.00 ± 5.54 a	93.10 ± 4.81 bcde	94.19 ± 1.52 c	118.40 ± 8.92 a	88.27 ± 4.04 b
7-day-old	3-day-old	Mated	0.80 ± 0.25 a	29.90 ± 9.73 a	65.00 ± 13.21 abc	77.20 ± 8.80 abc	135.10 ± 10.87 a	83.15 ± 9.29 ab
		Unmated	0.40 ± 0.16 a	13.00 ± 5.43 a	52.20 ± 7.40 ab	68.59 ± 10.75 ab	135.50 ± 20.85 a	72.01 ± 10.20 ab
	5-day-old	Mated	0.90 ± 0.43 a	19.30 ± 8.85 a	85.10 ± 12.08 abcd	66.80 ± 8.29 abc	151.80 ± 15.53 a	67.78 ± 8.39 ab
		Unmated	0.40 ± 0.16 a	16.90 ± 8.03 a	37.10 ± 6.50 a	58.81 ± 9.88 a	114.30 ± 21.22 a	53.96 ± 9.60 a
	7-day-old	Mated	0.80 ± 0.29 a	25.90 ± 9.27 a	103.80 ± 10.64 bcdef	91.52 ± 1.01 bc	157.90 ± 12.12 a	92.03 ± 1.36 b
		Unmated	0.44 ± 0.18 a	20.00 ± 8.36 a	85.78 ± 9.22 bcd	80.02 ± 9.67 abc	152.89 ± 12.87 a	81.10 ± 9.56 ab

Mean ± standard error followed by the same letter in the column do not differ by Turkey’s multiple-range test (*p* ≥ 0.05).

**Table 3:** The number of matings per cage, duration of matings per cage, number of eggs laid by a female per cage after pairing, percentage of eggs hatched per cage after pairing, total number of eggs laid by a female per cage, and total percentage of eggs hatched per cage from females in one of six physiological states paired with an unmated male within each age group in the laboratory.

Unmated Male	Female		Number of Matings per Cage	Duration of Matings per Cage (min)	Number of Eggs Laid by a Female per Cage after Pairing	Percentage of Eggs Hatched per Cage after Pairing (%)	Total Number of Eggs Laid by a Female per Cage	Total Percentage of Eggs Hatched per Cage (%)
3-day-old	3-day-old	Mated	0.50 ± 0.17 ab	15.80 ± 6.27 abcd	170.00 ± 14.54 g	91.25 ± 1.39 a	170.60 ± 14.71 e	90.99 ± 1.45 a
		Unmated	1.10 ± 0.10 cd	26.60 ± 3.93 bcdef	37.70 ± 14.95 abc	81.02 ± 10.30 a	37.70 ± 14.95 abc	81.02 ± 10.30 a
	5-day-old	Mated	0.20 ± 0.13 a	5.90 ± 3.93 ab	94.70 ± 9.75 defg	88.24 ± 3.19 a	119.90 ± 12.44 de	87.98 ± 2.46 a
		Unmated	1.30 ± 0.15 d	35.50 ± 4.27 cdef	124.90 ± 10.54 efg	98.00 ± 0.79 a	124.90 ± 10.54 de	98.00 ± 0.79 a
	7-day-old	Mated	0.40 ± 0.16 a	13.00 ± 5.43 abc	52.20 ± 7.40 abcde	68.59 ± 10.75 a	135.50 ± 20.85 de	72.01 ± 10.20 a
		Unmated	1.10 ± 0.10 cd	30.90 ± 2.96 cdef	94.00 ± 17.55 cdefg	86.90 ± 7.40 a	94.00 ± 17.55 cde	86.90 ± 7.40 a
5-day-old	3-day-old	Mated	0.50 ± 0.17 ab	22.90 ± 8.31 abcdef	170.70 ± 13.35 g	71.09 ± 10.43 a	170.80 ± 13.34 e	71.10 ± 10.43 a
		Unmated	1.60 ± 0.40 d	55.30 ± 12.92 f	93.00 ± 25.47 bcdef	95.95 ± 2.46 a	93.00 ± 25.47 bcde	95.95 ± 2.46 a
	5-day-old	Mated	0.10 ± 0.10 a	6.00 ± 6.00 a	107.30 ± 9.81 efg	89.44 ± 3.36 a	135.80 ± 10.24 de	87.67 ± 3.38 a
		Unmated	1.00 ± 0.00 abc	35.00 ± 4.69 cdef	25.67 ± 11.14 ab	76.63 ± 11.73 a	25.67 ± 11.14 ab	76.63 ± 11.73 a
	7-day-old	Mated	0.40 ± 0.16 a	16.90 ± 8.03 abcd	37.10 ± 6.50 abcd	58.81 ± 9.88 a	114.30 ± 21.22 de	53.96 ± 9.60 a
		Unmated	1.00 ± 0.00 abc	35.90 ± 4.07 cdef	68.10 ± 18.62 bcdef	81.41 ± 8.79 a	68.10 ± 18.62 abcd	81.41 ± 8.79 a
7-day-old	3-day-old	Mated	0.50 ± 0.17 ab	18.50 ± 6.27 abcde	131.90 ± 9.77 fg	91.73 ± 2.53 a	134.00 ± 9.61 de	90.15 ± 2.47 a
		Unmated	1.10 ± 0.10 cd	34.70 ± 2.26 cdef	128.30 ± 21.51 efg	96.48 ± 1.59 a	128.30 ± 21.51 de	96.48 ± 1.59 a
	5-day-old	Mated	0.20 ± 0.13 a	8.00 ± 5.54 ab	93.10 ± 4.81 defg	94.19 ± 1.52 a	118.40 ± 8.92 de	88.27 ± 4.04 a
		Unmated	1.50 ± 0.27 d	46.60 ± 6.5 6 ef	107.80 ± 19.42 defg	97.16 ± 1.20 a	107.80 ± 19.42 de	97.16 ± 1.20 a
	7-day-old	Mated	0.44 ± 0.18 ab	20.00 ± 8.36 abcde	85.78 ± 9.22 cdefg	80.02 ± 9.67 a	152.89 ± 12.87 e	81.10 ± 9.56 a
		Unmated	1.00 ± 0.00 abc	42.33 ± 6.68 def	13.00 ± 4.18 a	77.56 ± 7.90 a	13.00 ± 4.18 a	77.56 ± 7.90 a

Mean ± standard error followed by the same letter in the column do not differ by Turkey’s multiple-range test (*p* ≥ 0.05).

**Table 4:** The number of matings per cage, duration of matings per cage, number of eggs laid by a female per cage after pairing, percentage of eggs hatched per cage after pairing, total number of eggs laid by a female per cage, and total percentage of eggs hatched per cage from females in one of six physiological states paired with a mated male within each age group in the laboratory.

Mated Male	Female		Number of Matings per Cage	Duration of Matings per Cage (min)	Number of Eggs Laid by a Female per Cage after Pairing	Percentage of Eggs Hatched per Cage after Pairing (%)	Total Number of Eggs Laid by a Female per Cage	Total Percentage of Eggs Hatched per Cage (%)
3-day-old	3-day-old	Mated	0.50 ± 0.31 abcd	12.90 ± 7.16 abcd	141.10 ± 12.31 bc	94.38 ± 1.04 b	148.20 ± 14.22 b	94.08 ± 1.10 b
		Unmated	2.40 ± 0.45 ef	63.20 ± 11.90 ef	135.80 ± 18.28 abc	61.73 ± 10.81 ab	135.80 ± 18.28 ab	61.73 ± 10.81 ab
	5-day-old	Mated	0.10 ± 0.10 a	3.70 ± 3.70 a	89.90 ± 14.29 abc	90.53 ± 2.34 b	119.20 ± 14.98 ab	75.67 ± 6.86 ab
		Unmated	2.50 ± 0.56 f	68.40 ± 15.49 f	134.90 ± 14.37 abc	78.32 ± 8.94 ab	134.90 ± 14.37 b	78.32 ± 8.94 ab
	7-day-old	Mated	0.80 ± 0.25 abcdef	29.90 ± 9.73 abcdef	65.00 ± 13.21 ab	77.20 ± 8.80 ab	135.10 ± 10.87 b	83.15 ± 9.29 ab
		Unmated	1.30 ± 0.15 cdef	36.80 ± 5.61 bcdef	100.80 ± 13.67 abc	62.29 ± 11.88 ab	100.80 ± 13.67 ab	62.29 ± 11.88 ab
5-day-old	3-day-old	Mated	0.30 ± 0.15 abc	11.60 ± 6.57 abc	171.00 ± 12.75 c	92.15 ± 1.89 b	175.10 ± 12.61 b	89.87 ± 1.74 b
		Unmated	1.00 ± 0.19 abcdef	31.50 ± 5.45 abcdef	91.25 ± 10.16 abc	82.74 ± 3.67 b	91.25 ± 10.16 ab	82.74 ± 3.67 b
	5-day-old	Mated	1.20 ± 0.47 abcdef	52.70 ± 30.46 abcdef	120.90 ± 7.64 abc	81.58 ± 6.91 b	161.60 ± 11.62 b	82.06 ± 5.41 b
		Unmated	1.80 ± 0.36 ef	50.60 ± 9.85 def	144.40 ± 20.60 bc	84.76 ± 5.10 b	144.40 ± 20.60 b	84.76 ± 5.10 b
	7-day-old	Mated	0.90 ± 0.43 abcde	19.30 ± 8.85 abcde	85.10 ± 12.08 abc	66.80 ± 8.29 ab	151.80 ± 15.53 b	67.78 ± 8.39 ab
		Unmated	1.60 ± 0.31 def	47.30 ± 11.81 cdef	69.10 ± 19.91 a	49.96 ± 14.00 a	69.10 ± 19.91 a	49.96 ± 14.00 a
7-day-old	3-day-old	Mated	0.22 ± 0.15 ab	6.33 ± 4.26 ab	129.56 ± 19.79 abc	88.31 ± 5.85 b	134.44 ± 20.10 ab	87.96 ± 5.40 b
		Unmated	1.33 ± 0.37 bcdef	33.11 ± 8.20 abcdef	125.44 ± 18.88 abc	79.03 ± 7.79 b	125.44 ± 18.88 ab	79.03 ± 7.79 b
	5-day-old	Mated	0.40 ± 0.22 abc	15.10 ± 8.29 abcd	89.90 ± 14.38 abc	62.50 ± 11.15 ab	118.40 ± 18.80 ab	59.81 ± 10.13 ab
		Unmated	1.20 ± 0.13 cdef	39.70 ± 4.00 cdef	114.10 ± 18.45 abc	83.81 ± 6.85 b	114.10 ± 18.45 ab	83.81 ± 6.85 b
	7-day-old	Mated	0.80 ± 0.29 abcde	25.90 ± 9.27 abcdef	103.80 ± 10.64 abc	91.52 ± 1.01 b	157.90 ± 12.12 b	92.03 ± 1.36 b
		Unmated	1.17 ± 0.40 abcdef	34.67 ± 13.33 abcdef	121.00 ± 22.86 abc	76.21 ± 8.38 ab	121.00 ± 22.86 ab	76.21 ± 8.38 ab

Mean ± standard error followed by the same letter in the column do not differ by Turkey’s multiple-range test (*p* ≥ 0.05).

## Data Availability

The data presented in this study are available on request from the corresponding author.
